# Cardiac phenotype in mouse models of systemic autoimmunity

**DOI:** 10.1242/dmm.036947

**Published:** 2019-03-08

**Authors:** Chandan Sanghera, Lok Man Wong, Mona Panahi, Amalia Sintou, Muneer Hasham, Susanne Sattler

**Affiliations:** 1 National Heart and Lung Institute, Imperial College London, London, W12 0NN, UK; 2 The Jackson Laboratory, 600 Main Street, Bar Harbor, ME 04609, USA

**Keywords:** Heart disease, Heart failure, Mouse model, Myocarditis, SLE, Systemic autoimmunity

## Abstract

Patients suffering from systemic autoimmune diseases are at significant risk of cardiovascular complications. This can be due to systemically increased levels of inflammation leading to accelerated atherosclerosis, or due to direct damage to the tissues and cells of the heart. Cardiac complications include an increased risk of myocardial infarction, myocarditis and dilated cardiomyopathy, valve disease, endothelial dysfunction, excessive fibrosis, and bona fide autoimmune-mediated tissue damage by autoantibodies or auto-reactive cells. There is, however, still a considerable need to better understand how to diagnose and treat cardiac complications in autoimmune patients. A range of inducible and spontaneous mouse models of systemic autoimmune diseases is available for mechanistic and therapeutic studies. For this Review, we systematically collated information on the cardiac phenotype in the most common inducible, spontaneous and engineered mouse models of systemic lupus erythematosus, rheumatoid arthritis and systemic sclerosis. We also highlight selected lesser-known models of interest to provide researchers with a decision framework to choose the most suitable model for their study of heart involvement in systemic autoimmunity.

## Abstract (precis)

**Summary:** Cardiac complications are common in systemic autoimmunity, and mouse models are essential for mechanistic and therapeutic studies. Here, the authors present a decision framework to facilitate model choice for selected cardiac phenotypes in systemic autoimmunity.

## Introduction

**Fig. 1.**
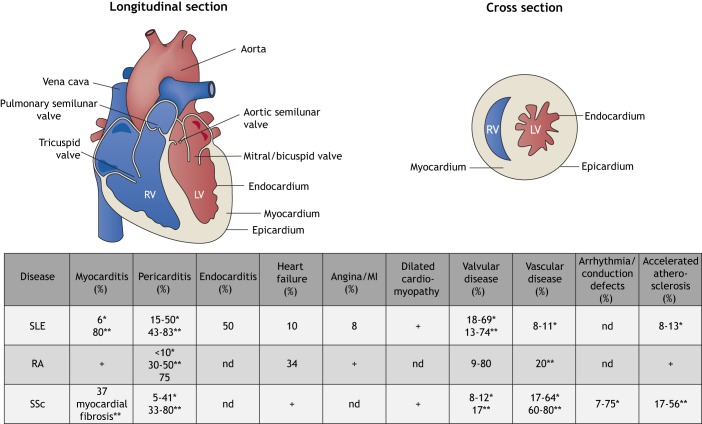
**Heart structures and their involvement in systemic autoimmune diseases such as systemic lupus erythematosus (SLE), rheumatoid arthritis (RA) and systemic sclerosis (SSc).** Numbers in the table indicate frequency of the manifestation in each disease as reported in the literature cited in this article. *, detected clinically due to patient presenting with symptoms; **, detected at post-mortem investigation in asymptomatic patients; +, presence reported without information available on exact incidence; nd, information not available in cited literature; RV, right ventricle; LV, left ventricle.

Aberrant activation of the immune system by self-antigens can lead to systemic autoimmune diseases such as systemic lupus erythematosus (SLE), rheumatoid arthritis (RA) and systemic sclerosis (SSc), and results in autoimmune-mediated tissue destruction. Autoimmune and cardiovascular diseases generally affect distinct demographic groups, yet patients suffering from systemic autoimmunity are at increased risk of developing cardiac complications (Jastrzębska et al., 2013). While increased cardiovascular morbidity and mortality in autoimmune patients have previously been attributed to accelerated atherosclerosis (Abou-Raya and Abou-Raya, 2006), we now appreciate that increased systemic inflammation and anti-heart auto-reactivity also directly affect cardiac cells and tissues (Knockaert, 2007). Fig. 1 shows the heart structures affected by SLE, RA and SSc, and the frequency of complications involving these structures.

**Fig. 2.**
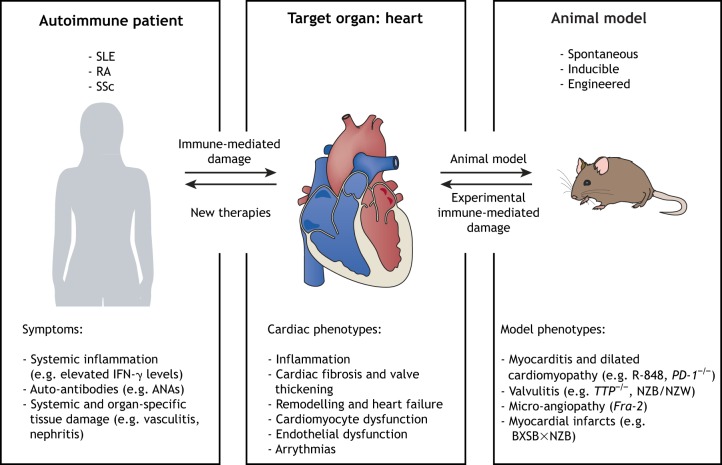
**Systemic autoimmune diseases, such as systemic lupus erythematosus (SLE), rheumatoid arthritis (RA) and systemic sclerosis (SSc) cause immune-mediated damage to the heart, which may manifest as acute inflammation, fibrosis, valve disease, remodelling towards heart failure, endothelial and cardiomyocyte dysfunction or arrhythmias.** To study selected or combined cardiac phenotypes, the research community benefits from a wide range of spontaneous, inducible and engineered mouse models, which allow mechanistic studies to improve our understanding and identify targets for new therapeutic approaches. IFN-γ, interferon gamma; ANAs, anti-nuclear antibodies; R-848, resiquimod; *PD-1*^−/−^, programmed cell death 1 knockout mice; *TTP*^−/−^, tristetraprolin knockout mice; *Fra-2*, fos-related antigen 2 transgenic mice.

Our understanding of the interplay between the cardiovascular and the immune system has seen a dramatic increase in recent years. This is largely due to the use of rodent models, which allow true mechanistic and cause-and-effect studies. Notably, however, animal models of autoimmune disease are rarely fully homologous to the human condition and generally only exhibit select features of the disease. It is further necessary to appreciate the cause of cardiac effects, which may include accelerated atherosclerosis, endothelial dysfunction, fibroblast activation, and autoantibody and cell-mediated autoimmune damage (see Box 1 for a glossary of terms). It is therefore crucial to choose the right model or combination of models for a selected phenotype or mechanism (Fig. 2).

**Box 1. Glossary**

**Alloantigens:** any antigen that is present only in some individuals of a species. Alloantigens stimulate the production of antibodies in individuals that do not have this antigen.

**Anichkov/Anitschkow cells:** enlarged mononuclear cells with ovoid nuclei, which are found in Aschoff bodies in inflamed hearts.

**Anti-La antibodies:** a type of anti-nuclear antibody, also called anti-Sjögren's-syndrome B (SSB) antibodies, associated with SLE and Sjögren's syndrome. Presence of anti-La antibody during pregnancy is correlated with congenital heart block.

**Anti-nuclear antibodies (ANAs):** autoantibodies against nuclear material, which is a hallmark of autoimmune diseases such as systemic lupus erythematosus (SLE).

**Anti-Ro antibody:** a type of anti-nuclear antibody, also called anti-Sjögren's-syndrome A (SSA) antibodies, targeting the self-proteins Ro60 and/or Ro52. They are commonly found in SLE and rheumatoid arthritis (RA).

**Anti-Smith antibody:** a type of anti-nuclear antibody against small nuclear ribonucleoproteins (snRNPs). Specific for SLE.

**Anti-topoisomerase-1/Scl70 autoantibodies:** anti-topoisomerase, also known as scleroderma antibody or anti-Scl-70 antibody, is an anti-nuclear antibody that targets self-DNA topoisomerase 1, commonly found in patients with autoimmune diseases such as systemic sclerosis (SSc).

**Aschoff bodies/nodules:** nodules of cellular infiltrate, connective tissue and dead heart cells found in the hearts of patients with rheumatic fever.

**Autoantibodies:** antibodies directed against the individual's own antigens.

**Autoimmune glomerulonephritis:** an autoimmune inflammatory disease that affects the kidney by damaging the glomeruli or the blood vessels in the kidneys, often a symptom in SLE.

**Bony ankylosis:** immobility or stiffness of a joint caused by the loss of articular cartilage, often associated with greater severity of RA.

**Cardiac valvulitis:** inflammation of the heart valves.

**Cardiomegaly:** enlargement of the heart.

**Complement C3:** the complement system is a key part of innate immunity and comprises numerous proteins that aid the removal of foreign or damaged cells. The hydrolysis of complement protein C3 can lead to a cascade of events that enhance opsonisation. An alternative pathway resulting from C3 cleavage allows the cleavage of downstream complement protein C5, which leads to recruitment of immune cells, an increase in vascular permeability and formation of membrane attack complex (MAC), causing target cells rupture.

**Diffuse disease:** a general term to describe diseases that are not occurring in a specific location (focal); rather, it involves a larger area.

**Dilated cardiomyopathy:** a cardiac condition where the pumping efficiency of the heart has decreased because the muscle layer of the heart is stretched and thinned.

**Effusion:** the build up of excess fluid in the chest or the lungs that can cause breathing difficulties and chest pain.

**Emphysema-like lung pathology:** emphysema is a member of a group of lung diseases called chronic obstructive pulmonary disease (COPD), which manifests itself as a shortness of breath. The elastic tissue in the lung is compromised; therefore, the air sacs in the lungs of these patients are over-inflated during inhalation. Furthermore, the bronchioles of the lungs also collapse, making gas exchange more challenging. Damage to capillaries of the lungs also reduces blood flow, making it more difficult to receive oxygen.

**Fibrillin 1:** an extracellular matrix glycoprotein and an essential component of microfibrils, which are found in both elastic and rigid structures (e.g. blood vessels, muscles and bones).

**Fibrinoid:** fibrinoid is a fibrin-like structure that is formed in the walls of vessels and connective tissues.

**Focal haemorrhage:** bleeding in a confined and specific location.

**Fractional shortening:** a measure of the contractility of the heart. Measured by the fraction of reduction of diastolic (maximum relaxation) dimension that occurs at systole (maximum contraction).

**Freund's complete adjuvant (FCA):** the water-in-oil adjuvant comprises heat-killed *Mycobacterium tuberculosis* in paraffin oil and mannide-mono-oleate. Used to boost the immune response at the site of antigen deposition to ensure efficient vaccination.

**Glomerular mesangial thickening:** the mesangium is the structure between the vessels inside the kidney glomerulus, surrounding capillaries and smooth muscle cells of the arterioles. Thickening of this layer is associated with membrano-proliferative glomerulonephritis, a type of kidney disease common in SLE and RA.

**Granuloma:** localised nodular inflammations formed by immune cells walling off foreign substances or areas of necrotic tissue.

**Hydroxychloroquine (HCQ):** orally administrated pharmaceutical treatment for RA and SLE that changes the pH in lysosomes, thus suppressing immune cell function.

**Hyperplasia:** enlargement in tissue size due to an increase in cell proliferation, resulting in a higher than normal cell number.

**Interstitial and perivascular fibrosis:** in the heart, interstitial fibrosis refers to the accumulation of collagen in the spaces between cardiomyocytes, while perivascular fibrosis indicates fibrosis around a blood vessel in the heart.

**Libman–Sacks endocarditis:** a form of endocarditis associated with SLE. Endocarditis is the inflammation of the inner layer of the heart, often also the mitral valve. The disease causes lesions (vegetations) in the tissue and haematoxylin bodies containing autoantibodies and degraded nuclear material.

**Lymphadenopathy:** enlarged lymph node.

**Major histocompatibility complex** **(MHC) class II I-A^g7^ and I-A^q^ haplotypes:** haplotype refers to the specific variation of a set of genes that are inherited together. A heterozygous individual will have two MHC haplotypes, one from each parent. In mouse, various MHC class II haplotypes exist: I-A^b^, I-A^d^, I-A^p^, I-A^q^, I-A^k^, I-A^r^, I-A^f^, I-A^s^ and I-A^g7^.

**Microangiopathy:** also known as microvascular disease; a disease of small blood vessels that can occur throughout the body.

**Monoclonal gammopathy:** a condition in which plasma cells produce an excess amount of monoclonal protein (M protein). M proteins are fragments of immunoglobulin generated by the abnormal proliferation of a plasma cell, generating clones of the same structure and therefore affinity to a particular epitope. This causes a shift in the size distribution of antibodies and can impair immune function.

**Monocytosis:** elevated monocyte levels in the blood.

**Myocardial angiostatin:** angiostatin is an angiogenesis inhibitor that blocks vessel growth; works by hindering endothelial cell proliferation.

**Pannus:** a fibrovascular structure that covers tissue in response to inflammation. It consists of macrophages, fibroblast-like mesenchymal cells and cells that secrete collagenolytic enzymes. Commonly found over a joint (in RA) or cornea.

**Pericarditis:** inflammation of the pericardium, the fibrous membrane that surrounds the heart.

**Polyarthritis:** an inflammatory disease in which at least five joints are affected simultaneously.

**Pristane:** a mineral oil originally derived from shark liver oil. Now, it can be synthesised, and the hydrocarbon compound is commonly used as an adjuvant for inducing tumours, arthritis and lupus nephritis in rodent models by stimulating antibody production.

**Quilty-like lesions:** tissue lesions that suggest the infiltration of long-lived lymphocytes into the endomyocardium, commonly found in allogeneic cardiac grafts.

**Regurgitation:** leakage or reverse flow of blood through the valves into the heart due to valve disease.

**Rheumatic carditis:** a side effect of acute rheumatic fever, which is a systemic inflammatory disease that causes the body to react to cardiac self-antigen, causing inflammatory lesions in the heart.

**Rheumatoid factors (RFs):** antibodies that target the Fc portion of immunoglobulin (Ig)G, often found in the blood of patients with RA.

**Semi-allogeneic:** allogeneic describes cells or tissue from a genetically different origin of the same species. Semi-allogeneic denotes individuals that share some genetic information, such as parents and offspring.

**Splenomegaly:** enlargement of the spleen.

**Synovial inflammation:** inflammation of the synovial membrane in the joints.

**Thrombosis:** formation of a blood clot in a blood vessel.

**Thymic atrophy:** the decrease in size of the thymus, a primary lymphoid organ crucial for T cell maturation in early life. The size of the thymus reduces with age naturally, where the stroma is replaced with fat tissue. As a hallmark of immune system senescence, thymic atrophy is related to impaired resistance to infection and increased susceptibility to cancer.

**Vasculitis:** inflammation of blood vessels that leads to their destruction.

A range of well-established mouse models of SLE, RA and SSc are available and commonly used in the immunological community. Yet, while the need to better understand, diagnose and treat cardiac involvement in systemic autoimmunity has become apparent, cardiac effects were rarely the focus of studies using these models and data on cardiac involvement is surprisingly scarce.

## Systemic lupus erythematous

SLE is a multisystem autoimmune disorder with diverse clinical features, including arthritis and haematological, cutaneous, renal and neurological manifestations (Kaul et al., 2016). It most commonly affects women and its development is influenced by a combination of genetic, environmental and hormonal factors (Lisnevskaia et al., 2014). SLE is characterised by the presence of anti-nuclear antibodies (ANAs), including anti-Smith (anti-Sm), anti-double-stranded-DNA (anti-dsDNA), anti-Ro and anti-La (Box 1) autoantibodies (Han et al., 2015). Triggers, including apoptotic cell debris, activate Toll-like receptors (TLRs) on plasmacytoid dendritic cells (pDCs) and induce excessive type I interferon (IFN) production. This causes immune system dysregulation and promotes antigen presentation to T cells (Crow et al., 2015; Kaul et al., 2016). T cells derived from SLE patients show persistent upregulation of co-stimulatory molecules, leading to increased activation and differentiation of autoantibody-producing B cells (Koshy et al., 1996). Likewise, B cell regulation is impaired, augmenting the production of autoantibodies and cytokines and promoting complement activation, ultimately resulting in tissue damage via immune-complex deposition (Kaul et al., 2016).

**Fig. 3.**
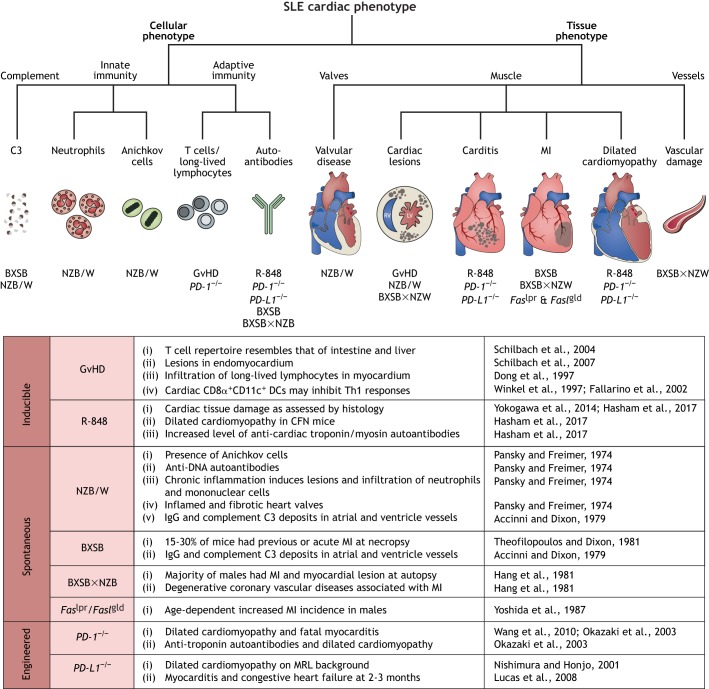
**Cardiac phenotypes in mouse models of systemic lupus erythematosus (SLE).** We include a decision tree to aid model choice based on the cellular mechanisms or tissue effect of interest, with a summary of the cardiac information available for each model. C3, complement-component 3 protein; GvHD, graft-versus-host disease; PD-1, programmed cell death 1; PD-L1, programmed death-ligand 1; R-848, resiquimod; lpr, lymphoproliferation; gld, generalized lymphoproliferative disease; DCs, dendritic cells; Th1, T-helper cell type 1; MI, myocardial infarction; RV, right ventricle; LV, left ventricle; MRL, Murphy Roths Large.

Cardiac involvement in patients with SLE can affect all components of the cardiovascular system (Fig. 1). A systematic review of 28 studies concluded that the risk of cardiovascular disease in SLE patients is at least double compared with the general population, and is one of the major causes of death (Manger et al., 2002; Schoenfeld et al., 2013). Male SLE patients also have nearly a 4-fold increased risk of cardiovascular disease compared to females (Pons-Estel et al., 2009; Urowitz et al., 2010). The most common cardiac complication of SLE is pericarditis (Box 1) (Buppajamrntham et al., 2014), followed by myocarditis (Thomas et al., 2017), which may develop into dilated cardiomyopathy (Box 1) and heart failure (Eriksson and Penninger, 2005). While the exact pathogenesis of SLE-associated pericarditis is still being explored, Bidani et al. demonstrated the deposition of immunoglobulin (Ig), complement C1q and complement C3 (Box 1) in pericardial vessel walls, accompanied by mononuclear cell infiltration (Bidani et al., 1980). SLE-associated myocarditis is likely mediated by immune-complex deposition, which leads to complement activation, inflammation and myocardial injury (Jain and Halushka, 2009). Up to 70% of SLE patients have a valvular abnormality, the most common being left-sided valve thickening and regurgitation (Box 1) (Omdal et al., 2001; Jensen-Urstad et al., 2002). Approximately 10% of SLE patients develop valvular disease from Libman–Sacks endocarditis (Box 1) (Moyssakis et al., 2007). SLE is also associated with an increased risk of coronary artery disease (CAD), which is linked to inflammation and endothelial dysfunction (Jain and Halushka, 2009). We discuss below the cardiac phenotyping data available for inducible, spontaneous and engineered mouse models of SLE. A decision tree to facilitate model choice is presented in Fig. 3.

### Inducible SLE models

Mice that develop graft-versus-host disease (GvHD) induced by semi-allogeneic (Box 1) cell transfer are a classical inducible SLE model for which information on cardiac effects is available. Researchers recently developed another SLE model, in which the TLR-7 agonist resiquimod (R-848) induces autoimmunity as a result of severe systemic inflammation and tissue damage. Although the pristane (Box 1)-induced-autoimmunity mouse model (Satoh and Reeves, 1994) is a classical model of both SLE and RA, we do not discuss it in this Review due to the lack of cardiac involvement data.

#### GvHD

In the ‘parent into F1’ GvHD model, parental donor T cells are transferred into semi-allogeneic F1 recipients, causing the donor T cells to react to host alloantigens (Box 1) (Perry et al., 2011). Chronic GvHD progresses to systemic autoimmunity and most prominently affects the skin, liver and intestine (Via, 2010).

Strikingly, while the heart does not appear grossly affected, T cells do infiltrate the heart and the cardiac T cell repertoire replicates that of affected organs, such as the intestine and the liver (Schilbach et al., 2004). Histological examination of the heart in chronic GvHD mice showed ‘Quilty-like’ lesions (Box 1) in the endomyocardium (Schilbach et al., 2007), which indicate infiltration of long-lived lymphocytes (Dong et al., 1997). Unlike in the intestines, however, no evidence of immune-mediated tissue destruction was observed in the hearts, despite increased expression of inflammatory cytokines. The presence of cardiac CD8α^+^CD11c^+^ dendritic cells (DCs) around the ‘Quilty-like’ lesions in the heart, but not in the intestine, has been suggested as a reason for this striking difference. CD8α^+^CD11c^+^ DCs inhibit the induction of inflammatory T cell responses (Winkel et al., 1997; Fallarino et al., 2002) and may thus prevent cardiac tissue destruction. Additional information on the cells and the factors involved in protecting the heart in these GvHD models would provide a crucial starting point to understand which tissue microenvironment conditions lead to the breakdown of this DC-mediated protection.

#### Resiquimod

TLR-7 activation via epicutaneous treatment with the TLR-7 agonists imiquimod or resiquimod induces SLE-like systemic autoimmune disease in mice of different genetic backgrounds (Yokogawa et al., 2014). This is in line with the observation that altered TLR signalling and interferonopathy contribute to the development of SLE in human patients and murine models (Christensen and Shlomchik, 2007; Ewald and Barton, 2011).

Our group recently used resiquimod to induce autoimmune disease in CFN mice (a strain obtained by crossing C57Bl6/J, FVB/NJ and NOD/ShiLtJ parental mice). We characterised cardiac disease and found a striking phenotype progressing from acute myocarditis to dilated cardiomyopathy (Hasham et al., 2017). Resiquimod-treated CFN mice developed dilated cardiomyopathy, and histopathological analysis revealed inflammatory damage to the cardiac tissue, especially in the endocardium, myocardium and papillary muscles, with features that resemble autoimmune pancarditis. Furthermore, we found increased levels of IgG2a and IgG2b autoantibodies against cardiac myosin and troponin. Both IgG2a and IgG2b have been associated with pathological immune responses in autoimmune disease (Ehlers et al., 2006). The resiquimod model therefore seems a time- and resource-efficient model to study cardiac involvement in systemic autoimmunity.

### Spontaneous SLE models

BXSB, Murphy Roths Large (MRL/1) and New Zealand black (NZB)×New Zealand white (NZW) (NZB/W) F1 mice spontaneously develop SLE-like disease manifestations (Celhar and Fairhurst, 2017). Importantly, all three strains also develop immune-complex-mediated lesions in coronary vessels and have an increased incidence of spontaneous myocardial infarcts (MIs). Infarcted areas are characterised by cardiomyocyte necrosis, focal haemorrhage (Box 1), leukocyte and macrophage infiltration, and scar tissue formation. IgG and complement C3 deposits were found in the vessels of both the atria and ventricles of all three strains, leading to the conclusion that immune-complex deposition plays a role in the underlying pathogenesis (Accinni and Dixon, 1979).

#### NZB/W

NZB/W F1 mice, obtained by breeding NZB females with NZW males, spontaneously develop SLE-like nephritis, haemolytic anaemia and classical SLE autoantibodies (Monneaux et al., 2001).

The cardiac phenotype of NZB/W mice has been thoroughly characterised and they are currently considered the gold standard for preclinical cardiac studies in SLE (Celhar and Fairhurst, 2017). Lesions containing Anichkov cells (Box 1) are evident in the epicardium, myocardium and endocardium from 4 months of age. Epicardial lesions consist of focal regions of mononuclear cell and neutrophil infiltrates, while myocardial lesions result from a more chronic inflammatory process and consist of mononuclear cell infiltrates and focal necrosis of myofibres. In the endocardium, both the subendocardial tissue and heart valves are affected, and the inflammatory lesions consist of mononuclear infiltrates, fibrinoid deposition and hyperplasia (Box 1). The valve leaflet is thickened and there is also evidence of fibrotic changes (Pansky and Freimer, 1974). Two recent studies used NZB/W F1 mice to assess the therapeutic benefit of hydroxychloroquine (HCQ; Box 1) in SLE. Long-term treatment resulted in reduced hypertension, reduced endothelial dysfunction, and less damage to the heart and kidneys. However, anti-dsDNA antibody levels remained unchanged, reflecting no change in SLE disease activity (Gomez-Guzman et al., 2014; Virdis et al., 2015). This suggests that the protective effects of HCQ are not related to blocking autoantibody-mediated damage, but a consequence of other mechanisms, such as decreased production of reactive oxygen species (ROS) and increased nitric oxide bioavailability (Meng et al., 1997; Gomez-Guzman et al., 2014). However, despite not targeting anti-dsDNA antibodies, the HCQ-mediated positive effects on endothelial dysfunction may make HCQ a useful future therapeutic to improve cardiac complications in SLE patients.

#### BXSB

BXSB mice are derived from a cross between a C57BL/6J female and a SB/Le F1 male (Murphy and Roths, 1978; Theofilopoulos and Dixon, 1981; Maibaum et al., 2000). They spontaneously develop an SLE-like disease including lymph node hyperplasia, immune-complex-mediated (autoimmune) glomerulonephritis (Box 1), thymic atrophy (Box 1), monocytosis (Box 1), elevated immunoglobulin concentrations associated with monoclonal gammopathy (Box 1), and moderately elevated ANAs and anti-ssDNA and anti-dsDNA antibodies (Theofilopoulos and Dixon, 1981). Notably, males show accelerated disease onset due to the mutant Y-linked autoimmune accelerator (*Yaa*) locus (Fossati et al., 1995). At necropsy, 15-30% of BXSB mice were found to have experienced a previous and/or acute MI that involved both ventricles and was extensive enough to be a possible contributing factor to death (Accinni and Dixon, 1979). However, this study currently appears to be the only one exploring cardiac involvement in this model.

#### BXSB×NZW

A hybrid model of BXSB males and NZW females shows an SLE phenotype including autoantibody production, circulating immunoglobulin-bound glycoprotein gp70 immune-complexes, and deposition of immunoglobulin and gp70 in glomeruli. Male F1 offspring develop a disease resembling the accelerated SLE phenotype that occurs in BXSB males (Hang et al., 1981).

The majority of these mice show degenerative coronary vascular disease associated with MI, with most males exhibiting myocardial lesions at necropsy. The disease showed delayed onset and oestrogen dependence in females, and necropsy revealed infarcts in only one third of females. The majority of vascular and myocardial lesions occurred in the right ventricle, right atrium and left subendocardium (Yoshida et al., 1987). However, coronary disease in BXSB×NZW mice does not seem to be immune-complex-mediated or associated with thrombosis (Box 1), as the vascular lesions did not present with an inflammatory response (Hang et al., 1981).

#### *Fas*^lpr^ (*lpr*) and *Fasl*^gld^ (*gld*)

Lymphoproliferation (*lpr*) and generalized lymphoproliferative disease (*gld*) mice carry defects in the apoptosis-mediating cell-surface molecule Fas and its ligand, Fasl, respectively. These mice develop lymphadenopathy (Box 1) because of an age-dependent accumulation of non-malignant CD4^–^CD8^−^ T cells in peripheral lymphoid organs. The *Fasl*^gld^ strain was discovered as an autosomal recessive mutation in C3H/HeJ mice (Roths et al., 1984). The *Fas*^lpr^ mutation was discovered during inbreeding of MRL/Mp mice derived from crosses between several parental strains – the LG/J strain with minor contributions from C3H/Di, C57BL/6 and AKR/J (Theofilopoulos and Dixon, 1985). The MRL/Mp background itself carries a normal *Fas* gene but still develops an SLE-like autoimmune disorder later in life (Perry et al., 2011).

While the heart remains largely unaffected in MRL-*Fas*^lpr^ mice (Theofilopoulos and Dixon, 1981), there seems to be an age-dependent increase in MI incidence in males, albeit at lower rates than in BXSB×NZW mice (Yoshida et al., 1987).

### Engineered SLE models

A variety of engineered mouse models exhibit phenotypes similar to selected SLE manifestations. Notably, most of these models were not developed with the intention to induce autoimmunity. However, any disruption of pathways involved in the resolution of inflammation and feedback inhibition has the potential to cause aberrant immune activation and autoimmunity. The most relevant model with both an autoimmune and cardiac phenotype is based on the disruption of the programmed cell death-1 (PD-1)/PD-1 ligand 1 (PD-L1) axis.

#### PD-1 and PD-L1

PD-1 and its ligand, PD-L1, are potent negative regulators of T cell activation. They have been implicated in various autoimmune conditions and have recently obtained notoriety due to their clinical use in cancer therapy. Despite their success in oncology, PD-1 checkpoint inhibitors have also caused severe cardiac side effects such as myocarditis (Varricchi et al., 2017). Importantly, PD-L1 is expressed in cardiac tissue (Nishimura and Honjo, 2001). While disruption of PD-1/PD-L1 signalling induces SLE-like autoimmune disease with dominant cardiac involvement (Nishimura and Honjo, 2001; Nishimura et al., 2001), the relative contributions of the direct effects of PD-L1 depletion in the heart versus the indirect effects mediated by systemic autoimmunity are not yet understood and need to be carefully considered.

PD-1 deficiency in mice causes dilated cardiomyopathy, which is correlated with autoantibodies against cardiac troponin I, and the development of fatal myocarditis (Wang et al., 2010; Okazaki et al., 2003).

PD-L1-deficient mice develop dilated cardiomyopathy (Nishimura and Honjo, 2001). A particularly interesting study investigated PD-L1 depletion in the autoimmune-prone MRL-*Fas*^lpr^ background. Survival of *PD-L1*^−/−^ MRL-*Fas*^lpr^ mice was significantly decreased compared to the MRL-*Fas*^lpr^ controls. While MRL-*Fas*^lpr^ mice develop lymphadenopathy, splenomegaly (Box 1), skin lesions and fatal renal disease at around 5-6 months of age, *PD-L1*^−/−^ MRL-*Fas*^lpr^ mice developed congestive heart failure resulting from autoimmune myocarditis as early as 2-3 months of age. Histopathological analysis revealed pancarditis spanning the endocardium, myocardium, epicardium, atria and both ventricles. Macrophages, followed by T cells, accumulated in the hearts and high titres of autoantibodies against cardiac myosin and cardiac troponin I were present at 2 months of age. Autoantibodies were detected only when overt disease was present, suggesting that they may not be involved in the initial development of heart disease, and titres of anti-dsDNA autoantibodies were not increased over control levels. Notably, *PD-L1*^−/−^ mice on both the MRL^+/+^ and MRL-*Fas*^lpr^ background developed a very similar degree of myocarditis, showing that the *Fas*^lpr^ mutation does not have a role in the development of autoimmune-mediated heart disease under these conditions (Lucas et al., 2008).

## Rheumatoid arthritis

RA is a systemic autoimmune disorder characterised by synovial inflammation (Box 1), autoantibody production, and destruction of bone and cartilage. Systemic features of RA include cardiovascular, pulmonary, skeletal and psychological complications. RA is more common in women, and is associated with genetic and environmental risk factors (McInnes and Schett, 2011). Rheumatoid factors (RFs; Box 1) were the first type of autoantibody described to be associated with RA (Waaler, 1940) and their presence is used as a diagnostic biomarker (Whiting et al., 2010). They target the Fc region of IgG in immune-complexes and, under healthy conditions, serve an important physiological function through facilitating and clearing immune-complexes. In RA, RF-mediated enhancement of immune-complex formation potentiates the arthritogenicity of other pathological autoantibodies, including anti-citrul­linated protein antibodies (ACPAs) (Van Snick et al., 1978; Pope et al., 1974). ACPAs are IgG autoantibodies that self-aggregate into immune-complexes, activate complement, stimulate inflammation and cause chronic synovitis (Silverman and Carson, 2003). IgG immune-complexes also recruit T cells, thus perpetuating RA (Lang et al., 1999).

**Fig. 4.**
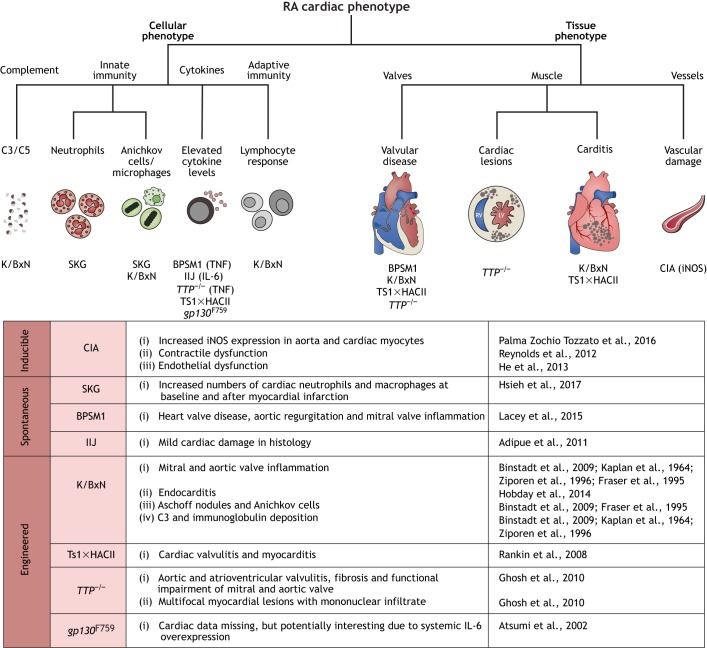
**Cardiac phenotypes in mouse models of rheumatoid arthritis (RA).** We include a decision tree to aid model choice based on the cellular mechanisms or tissue effect of interest, with a summary of the cardiac information available for each model. C3/C5, complement-component 3/5 protein; IIJ, inherited inflamed joint; IL-6, interleukin-6; TTP, tristetraprolin; TNF, tumour necrosis factor; CIA, collagen-induced arthritis; iNOS, inducible nitric oxide synthase overexpression.

Patients with RA are 30-60% more likely to suffer from cardiovascular disease compared to the general population, and this complication accounts for around half of all deaths in RA (Han et al., 2006; Avina-Zubieta et al., 2008). Furthermore, male patients with RA are significantly more likely to suffer from a cardiovascular event compared to female patients (Naranjo et al., 2008). One of the most common cardiac manifestations in RA is pericardial inflammation (Fig. 1), which is also a prognostic indicator of the severity of this autoimmune disease (Voskuyl, 2006). RA also increases the risk of vascular disease by causing changes in lipid handling (Maradit-Kremers et al., 2005) and instigating vasculitis (Box 1) in coronary vessels (Voskuyl, 2006), culminating in a higher incidence of asymptomatic ischemia and MI (Maradit-Kremers et al., 2005). The immunological mechanisms underlying the cardiac manifestations of RA are mostly attributed to the chronic inflammation with increased c-reactive protein (CRP), TNF, IL-1 and IL-6. A more atherogenic lipid profile with less-efficient high-density lipoprotein (HDL) cholesterol, and paradoxically fewer and smaller but denser and more pathological low-density lipoprotein (LDL) cholesterol, is prevalent (Castañeda et al., 2016) and accounts for a higher risk of ischemic cardiac disease. There may also be a degree of shared inflammatory mediators between joint inflammation and the extra-articular manifestations of RA. For example, ACPA targets, such as vimentin, enolase and fibronectin, are also present in the myocardium (Giles et al., 2012). Rheumatoid granulomas (Box 1) can accumulate in any organ, including the heart, and their location determines the related functional impairment (Voskuyl, 2006). Chronic inflammation also triggers the deposition of acute-phase protein amyloid, which can cause a range of pathologies, including conduction defects, cardiomegaly (Box 1), cardiomyopathy and heart failure (Voskuyl, 2006; Kuroda et al., 2006). The cardiac phenotyping data available for inducible, spontaneous and engineered mouse models of RA is discussed below. A decision tree to facilitate model choice is presented in Fig. 4.

### Inducible RA models

Immunisation with collagen is a common way to induce experimental RA. It is also the only inducible RA mouse model with any, albeit little, information available on potential cardiac involvement.

#### Collagen-induced arthritis (CIA)

Murine CIA closely resembles human RA, and develops upon immunisation with type II collagen in Freund's complete adjuvant (Box 1) (Courtenay et al., 1980; Asquith et al., 2009). Features of CIA include polyarthritis (Box 1), which is characterised by synovial hyperplasia, inflammatory infiltration, and cartilage and bone erosion. The development of CIA is related to B and T cell responses, including collagen-specific T cells and anti-collagen type II antibodies of the IgG2 isotype (Asquith et al., 2009).

Studies on the heart in CIA are rare and have focused on vascular effects. One study found that CIA increased inducible nitric oxide synthase (iNOS) expression in the aorta and in cardiac myocytes (Palma Zochio Tozzato et al., 2016), and a separate study reported endothelial contractile dysfunction (Reynolds et al., 2012). Given that the CIA model reproduces the underlying autoimmune mechanisms of human RA, these observations may reflect the vascular and cardiac changes that occur in RA patients, with studies also showing that iNOS expression contributes to endothelial dysfunction (Mäki-Petäjä et al., 2008).

### Spontaneous RA models

#### BALB/c *ZAP-70*^W163C^ mutants (SKG)

SKG mice harbour a spontaneous mutation in the protein kinase ZAP-70, which causes self-reactive T cells to escape negative selection in the thymus, thereby leading to T cell-mediated arthritis (Rothe et al., 2017; Guerard et al., 2016b). The arthrogenicity of the ZAP-70 mutation seems independent of genetic background (Guerard et al., 2016a). However, different environmental conditions, such as variation in the microbiome, might change the extra-articular manifestations (Rehaume et al., 2014). At baseline, SKG mice have increased numbers of cardiac neutrophils and monocytes/macrophages. Following MI, researchers observed a further 6-fold increase in infiltrating neutrophil numbers and a persistently high Ly-6C^+^ macrophage infiltrate (Hsieh et al., 2017). The change in the number and phenotype of cardiac immune cells is likely to negatively influence cardiac repair and ventricular remodelling, leading to worse outcomes. This is corroborated by the role of excessive numbers of infiltrating neutrophils in mediating tissue damage in patients post-MI (Carbone et al., 2013).

#### Bone phenotype spontaneous mutation 1 (BPSM1)

BPSM1 mutant mice carry a spontaneous mutation in the *Tnf* gene, which leads to constitutive overexpression of tumour necrosis factor (TNF) (Lacey et al., 2015). Excess levels of inflammatory cytokines, including TNF, are known to mediate RA pathogenesis (Parameswaran and Patial, 2010; Sakkou et al., 2018).

BPSM1 C57BL/6 mice develop polyarthritis and heart valve disease accompanied by aortic regurgitation and inflammation of the mitral valve (Lacey et al., 2015). Thus, these mice may be an interesting model to study the role of TNF in immune-mediated heart disease.

#### Inherited inflamed joints (IIJ)

The IIJ strain was developed by crossing an SJL/J wild-type male mouse that had spontaneously developed arthritis (Waldner et al., 2000) to SJL/J females. IIJ mice develop bone and cartilage erosion, synovial hyper-proliferation and immune cell infiltration in distal joints. The model also shows increased IL-6 and elevated serum antibody levels with systemic inflammation of multiple organs. This includes mild cardiac involvement, which was detected histologically. However, unlike in human RA patients, joint pathology is asymmetric and it remains to be determined whether the B cell expansion and the presence of IgG support the notion that the IIJ mouse truly models autoimmunity (Adipue et al., 2011).

### Engineered RA models

A variety of engineered mouse models show RA-associated phenotypes and have been valuable in the investigation of causative mechanisms ranging from antigen-specific adaptive T cell responses (the K/BxN and TS1×HACII mice) to increased systemic cytokine levels [in tristetraprolin (*TTP*)^−/−^ and *gp130*^F759^ mice]. Heart involvement has been described for the K/BxN model, but information for cardiac phenotypes in TS1×HACII mice is not available.

#### K/BxN T cell receptor (TCR) transgenic

K/BxN mice were generated by crossing the TCR transgenic KRN line with NOD mice expressing the major histocompatibility complex (MHC) class II molecule A^g7^ (Box 1) (Kouskoff et al., 1996). In these mice, T cells recognise glucose-6-phosphate isomerase (GPI) self-peptides presented by A^g7^. This activates GPI-reactive B cells, which leads to the production of anti-GPI autoantibodies, causing arthritis (Matsumoto et al., 1999). The model shares many clinical, histological and immunological features of human RA, and its earliest phenotypic manifestation is joint swelling at around 3 weeks of age. Other features include symmetrical articular involvement, leukocyte infiltration, synovitis, pannus formation (Box 1), and cartilage and bone erosion followed by remodelling and fibrosis. However, these mice do not have elevated RF and develop high titres of anti-GPI autoantibodies (Kouskoff et al., 1996; van Gaalen et al., 2004).

Several studies observed cardiac involvement in the K/BxN model. This includes inflammation of the mitral valve (Binstadt et al., 2009; Kaplan et al., 1964; Ziporen et al., 1996), and occasionally of the aortic valve (Binstadt et al., 2009; Fraser et al., 1995), endocarditis (Hobday et al., 2014), and myocardial Aschoff nodules (Box 1) and Anichkov cells (Binstadt et al., 2009; Fraser et al., 1995). Mitral valve inflammation correlated with complement C3 and Ig deposition as seen in patients with rheumatic carditis (Box 1) or Libman–Sacks endocarditis (Binstadt et al., 2009; Kaplan et al., 1964; Ziporen et al., 1996). Mice developed endocarditis at as early as 3 weeks of age. The inflammatory cells involved were mainly macrophages and T cells. However, separate mechanisms have been implicated in joint inflammation and endocarditis. While the initiation of both heart and joint inflammation involves the TCR, A^g7^ and B cells, further progression of disease differs in their dependence on complement and Fc receptors (Hobday et al., 2014; Binstadt et al., 2009; Fraser et al., 1995). While complement C5 is required for disease progression in the joints, endocarditis depends on Fc receptors and is independent of C5. This suggests that autoantibodies binding to the mitral valve might provoke inflammation via their interaction with activating Fc receptors.

#### TS1×HACII

TS1×HACII transgenic mice express hemagglutinin (HA) driven by an MHC class II promoter (and is thus expressed systemically by antigen-presenting cells), as well as a TCR that recognises HA. They develop spontaneous RA with symmetrical ankle and wrist swelling, mononuclear lymphocytic infiltration, cartilage and bone erosion, and pannus formation. The majority of the synovium-infiltrating cells are neutrophils, with a few T cells. Disease development in these mice is accompanied by inflammatory cytokine production, systemic B cell activation and an enhanced lymph node response. However, RA also develops in mice lacking B cells, indicating that autoantibody production is not required for disease initiation (Rankin et al., 2008). Heart involvement was also determined upon initial phenotyping of these mice, with some animals developing cardiac valvulitis (Box 1) and myocarditis (Rankin et al., 2008).

#### *TTP*^−/−^ mice

TTP is an RNA-binding protein that has anti-inflammatory effects via binding and destabilising *Tnfa* mRNA. This leads to TNF-excess syndrome characterised by systemic inflammation. *TTP*^−/−^ mice exhibit autoimmunity and their phenotypic features include arthritis, dermatitis, conjunctivitis, glomerular mesangial thickening (Box 1), loss of adipose tissue and myeloid hyperplasia (Taylor et al., 1996).

Mice as young as 7 weeks also develop aortic and left-atrioventricular valvulitis, which is likely related to the greater haemodynamic load in the left chambers. Some mice also exhibit small multifocal myocardial lesions with mononuclear infiltrates. Monocytes and granulocytes, but not lymphocytes, infiltrate the valves. Other features include valve fibrosis, gross thickening, elastic fibre disruption, collagen deposition and neovascularisation. *TTP*^−/−^ mice can also display left-ventricular and atrial enlargement in both end-systolic and end-diastolic phases, consistent with left ventricular overload and functional impairment of the mitral and aortic valve. However, left-ventricular fractional shortening (Box 1) and ejection fraction remain unaltered. The underlying pathogenesis has been attributed to increased local TNF levels, presumably produced by the infiltrating leukocytes. Notably, mice lacking TTP and both TNF receptors also had mild valve-leaflet thickening and cellular infiltration, suggesting that TTP may also cause TNF-independent valve inflammation (Ghosh et al., 2010). However, the valvular phenotype in these mice exhibits neither T nor B cell infiltration, thus does not closely resemble valvular disease in human RA, which is characterised by the presence of lymphocytic granulomas (Iveson et al., 1975).

#### *gp130*^F759^ (F759)

The F759 mouse line has been generated by targeted mutation to insert mutant human *IL6* carrying a tyrosine-to-phenylalanine substitution at position 759 (Y759F) into the corresponding region of the mouse gene. Y759F disrupts inhibitory SHP-2 signalling downstream of interleukin-6 (IL-6), and F759 mice spontaneously develop age-dependent chronic and progressive arthritis accompanied by autoantibody production and T cell abnormalities from around 1 year of age. Histologic examination showed leukocytes infiltrating the joint space, hyperplasia of the synovium with pannus formation, cartilage and bone destruction, and bony ankyloses (Box 1) (Atsumi et al., 2002).

IL-6 has been implicated as a cause of cardiac hypertrophy, and an increase in IL-6 levels is a risk factor for sudden cardiac death in patients with CAD (Hagiwara et al., 2007; Fisman et al., 2006). Although the cardiac phenotype of the F759 mouse line has not been studied yet, with increasing appreciation of the detrimental effects of increased systemic levels of inflammatory cytokines on the heart it might soon prove a valuable and relevant mouse model to study this aspect of heart disease.

## SSc

SSc, also termed scleroderma, is a rare systemic autoimmune condition associated with high mortality. Skin fibrosis is the distinguishing hallmark of the disease, and microvascular damage and generalised fibrosis in multiple organs are common (Allanore et al., 2015; Denton and Khanna, 2017). The primary underlying pathogenesis of the disease is thought to involve vascular injury, triggering endothelial cell and platelet activation, endothelin-1 and chemokine production, and increased expression of adhesion molecules. This leads to the recruitment and infiltration of inflammatory cells, including type 2 T helper cells (Th2), macrophages, pDCs and autoantibody-producing B cells, among others. Factors secreted from these cells, including TGF-β, IL-13 and IL-6, activate fibroblasts that can differentiate into myofibroblasts, resulting in excess extracellular matrix (ECM) production and fibrosis (Allanore et al., 2015). ANAs are present in 90% of patients, with the three major subclasses of autoantibodies in SSc being anti-centromere, anti-topoisomerase (Topo)-1 (Box 1) and anti-RNA polymerase III antibodies (Ho and Reveille, 2003). While different ANA profiles are associated with different types of SSc, anti-Topo-1 antibodies are the most common and are associated with pulmonary fibrosis and cardiac involvement (Hesselstrand et al., 2003).

These pathogenic processes in SSc, including diffuse lesions (Box 1) in the micro- and macro-vasculature, immune dysfunction, and fibrosis, damage the myocardium, endocardium, pericardium and conduction systems of the heart (Fig. 1) (Ferri et al., 2005). As such, a range of cardiac pathologies, including pericardial effusions (Box 1), conduction defects, ischemia and hypertension may occur (Kahan and Allanore, 2006; Agrawal et al., 2016). In the myocardium, patchy distribution of myocardial fibrosis and contraction band necrosis is pathognomonic of the disease (Lambova, 2014). However, myocardial involvement in SSc is distinct from atherosclerotic coronary disease as it may involve the subendocardial layer, and hemosiderin deposits are absent (Champion, 2008). Cardiac involvement is often underestimated in SSc, yet accounts for up to one-third of the mortality among SSc patients (Ferri et al., 2002; Steen and Medsger, 2000). While this may be close to the 25% mortality due to cardiac events in the general population, mortality in SSc patients occurs up to a decade earlier (Belch et al., 2008).

**Fig. 5.**
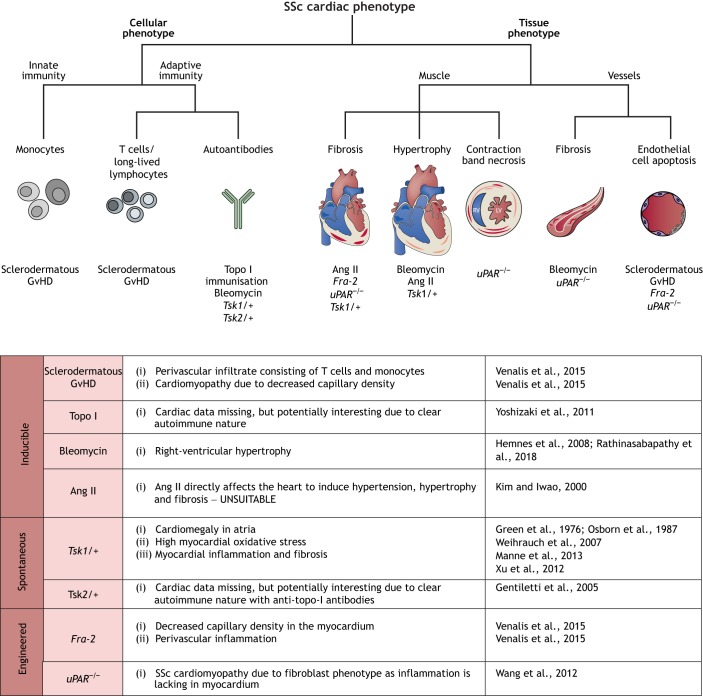
**Cardiac phenotypes in mouse** **models of systemic sclerosis (SSc).** We include a decision tree to aid model choice based on the cellular mechanisms or tissue effect of interest, with a summary of the cardiac information available for each model. GvHD, graft versus host disease; Topo I, type I topoisomerase; Tsk, tight skin; Ang II, angiotensin type II; Fra-2, fos-related antigen 2; uPAR, urokinase receptor.

Most currently available SSc models focus on fibrosis as the dominant phenotype, but may not replicate the underlying autoimmune aspect of the disease. The full range of available models has been reviewed elsewhere (Artlett, 2014). For the purpose of this Review, we considered which model may in fact have a systemic inflammatory/autoimmune component and we focus on those with established or potential immunological and cardiac relevance. A decision tree to facilitate model choice is presented in Fig. 5.

### Inducible SSc models

A wide range of inducible mouse models of SSc has been established, the majority of which focus on fibrosis of the skin and lung as their primary phenotypes. These models are based on different underlying principles, including the induction of autoimmunity (e.g. the anti-DNA Topo-I and GvHD models), tissue damage and inflammation followed by fibrosis (e.g. the hypochlorous and bleomycin models), or the modification of systemic hormone systems [e.g. the angiotensin (Ang) II model].

#### Sclerodermatous GvHD

The primary inducible model used to study human SSc is chronic GvHD, described above (see SLE section). Transfer of donor splenocytes and/or bone marrow to recipients matched for MHC but mismatched at loci encoding minor histocompatibility antigens (Beyer et al., 2010; Jaffee and Claman, 1983; Zhang et al., 2002) induces autoimmunity. A modified model based on the injection of splenocytes into recipients deficient in mature T and B cells exhibits all major components of human SSc, including dermal thickening, progressive fibrosis of internal organs, vasoconstriction and altered expression of vascularity markers in skin and internal organs, early immune activation, inflammation in skin and internal organs, and the production of anti-DNA Topo I/Scl70 autoantibodies (Box 1) (Ruzek et al., 2004).

When using chronic GvHD as an SSc model, researchers found that the animals developed cardiomyopathy associated with decreased capillary density in the myocardium, which is a hallmark of microangiopathy (Box 1) in human SSc patients. Increased perivascular inflammation due to infiltration of T cells and monocytes has also been observed (Venalis et al., 2015).

#### DNA Topo-I immunisation

Autoantibodies against Topo I are a central feature of SSc and correlate with dermal and pulmonary fibrosis, and, importantly, with cardiac involvement (Hesselstrand et al., 2003). Immunisation of mice with recombinant Topo I and Freud's complete adjuvant increased the levels of anti-Topo-I and other autoantibodies and induced dermal sclerosis and pulmonary fibrosis (Yoshizaki et al., 2011). Although there is no information available on cardiac pathology in this model, it seems a promising candidate to investigate cardiac involvement in SSc due to its underlying autoimmune nature.

#### Bleomycin

Bleomycin is an antineoplastic antibiotic used in cancer chemotherapy that can cause injury and fibrosis in the skin and internal tissues of mice (Yamamoto et al., 1999, 2000). Cardiac involvement in bleomycin-induced sclerosis has been investigated primarily as a knock-on effect of pulmonary fibrosis and lung pathology. Administration of bleomycin to induce pulmonary fibrosis also causes right-ventricular hypertrophy (Hemnes et al., 2008; Rathinasabapathy et al., 2018). Subsequent treatment with sildenafil, a phosphodiesterase type 5 inhibitor used to treat pulmonary arterial hypertension, decreased the degree of pulmonary, vascular and right-ventricle fibrosis as well as the associated cardiac hypertrophy. Notably, while the majority of bleomycin-induced SSc studies focus on inducing fibrosis as their main readout, bleomycin treatment does induce the release of autoantibodies, most likely in response to tissue damage (Ishikawa et al., 2009). It therefore remains to be established whether there is a role of anti-heart autoimmunity in the bleomycin model and whether this model is therefore suitable for investigating immune-mediated heart phenotypes in SSc.

#### Angiotensin II

Ang II has been proposed as an SSc model due to its potent fibrotic effect on the skin (Stawski et al., 2012). SSc patients have elevated serum levels of Ang II (Kawaguchi et al., 2004), and pharmacological inhibition of the Ang II receptor can ameliorate fibrosis in mice (Murphy et al., 2015). Notably, however, Ang II is the main effector of the renin-angiotensin system (RAS), which is also a key regulator of blood pressure and cardiac function. It is therefore also used in cardiovascular research to induce hypertension, cardiac hypertrophy and fibrosis (Kim and Iwao, 2000).

While Ang II mice might be a useful model for questions related to selected mechanisms and phenotypes in either SSc or cardiovascular pathology, it is likely to be unsuitable as a model for the heart disease that develops as a consequence of systemic autoimmunity.

### Spontaneous SSc models

The two main spontaneous mutations causing SSc-like manifestations in mice are the tight skin (*Tsk*)*1* and *Tsk2* mutations (Green et al., 1976). *Tsk1*/+ mice harbour a mutation in the fibrillin 1 (*Fbn-1*) gene (Box 1) (Siracusa et al., 1996), whereas *Tsk2*/+ mice have a mutation in the collagen type III, alpha I (*Col3a1*) gene (Long et al., 2014). Both mutations are homozygous lethal, so mice need to be maintained as heterozygotes.

#### The tight skin-1 (*Tsk1*/+) mouse

*Tsk1*/+ mice exhibit skin, tendon and cardiac fibrosis, and autoimmunity with extensive B cell activation and autoantibody formation, but also develop phenotypes that differ from human SSc, including hypodermal collagen accumulation (Baxter et al., 2005) and an emphysema-like lung pathology (Box 1) (Rossi et al., 1984). *Tsk1*/+ mice also lack several features of SSc such as vascular injury and mononuclear cell infiltration (Pablos et al., 2004), and show normal capillary density in myocardial tissue without changes in perivascular inflammation (Venalis et al., 2015).

Cardiac effects in *Tsk1*/+ mice were first reported in the original description of the *Tsk1*/+ model, which showed cardiomegaly most dominant in the atria (Green et al., 1976; Osborn et al., 1987). Heart enlargement was accompanied by a shift in the ratio of collagen I, II and III. In healthy hearts, collagen I accounts for 67% of total collagen. In *Tsk1*/+ mice this increases to 95%. Notably, the ECM isolated from the hearts of *Tsk1*/+ mice stimulates endothelial cells to become fibroblasts, which indicates a pro-inflammatory and fibrotic effect (Xu et al., 2011). Electron microscopy of the left ventricle further revealed that cardiomyocytes are present in a non-relaxed state, and showed accumulation of perivascular and interstitial oedema fluid, and isolated areas of cardiomyocyte necrosis (Box 1) (Osborn et al., 1987). *Tsk1*/+ mice also have high levels of myocardial oxidative stress, which increases myocardial angiostatin (Box 1) production and inhibits endothelium-dependent vasodilation (Weihrauch et al., 2007). Considering that ROS generation in human SSc drives the differentiation of cardiac fibroblasts into myofibroblasts, accounting for the increased production of ECM proteins such as collagen I and the resultant fibrosis, parallels can be drawn between this model and the human phenotype of SSc.

The hearts of *Tsk1*/+ males also have significantly more total collagen than those of wild-type males (Manne et al., 2013). Cardiac fibrosis in the *Tsk1*/+ mouse line was used to study the effects of D-4F, an apolipoprotein A-I mimetic that improves vascular function in SSc-related vascular problems affecting the heart (Weihrauch et al., 2007). This study demonstrated that D-4F treatment significantly reduces posterior-wall thickening, highlighting a reduction in fibrosis. It was also found that IRF5 is present in the hearts of untreated *Tsk1*/+ mice, which also had high levels of apoptotic cells. D-4F treatment decreased IRF5 expression, which could explain the reduction in inflammation and the reduced number of apoptotic cells in the treated animals compared to controls (Xu et al., 2012). These findings, combined with clinical trials affirming the role of D-4F in improving HDL function and reducing oxidative stress (Dunbar et al., 2017), support the use of D-4F and other IRF5-targeting agents as a potential therapeutic strategy to protect against SSc-induced cardiac inflammation. It should also be noted that immune cells, such as monocytes, myeloid cells and B cells, express IRF5 at much higher levels than the heart, meaning that the D-4F-induced changes in myocardial IRF5 expression could also be related to changes in myocardial immune cell content (Xu et al., 2012).

#### The tight skin-2 (*Tsk2*/+) mouse

The *Tsk2*/+ mouse strain has many features of human SSc, including fibrosis, ECM abnormalities and ANAs (Gentiletti et al., 2005; Long et al., 2014). Notably, *Tsk2*/+ mice have detectable levels of anti-Topo-I autoantibodies (Gentiletti et al., 2005), which are the most frequently observed autoantibodies in SSc patients that have developed pulmonary fibrosis and cardiac complications (Hesselstrand et al., 2003). As of now, there are no studies on cardiac phenotypes in the *Tsk2*/+ mouse strain. However, presence of anti-Topo-I autoantibodies may make them a suitable model for future studies.

### Engineered SSc models

A wide range of engineered SSc models exists. However, the majority have been developed by specifically targeting pathways involved in fibrosis or vascular function, and have no underlying inflammatory or auto-immune component. We identified *Fra-2* transgenic and *UPAR*^−/−^ mice as two potentially relevant models of autoimmunity. However, although the cardiac manifestations documented in these mice appear comparable to the phenotype observed in human patients, the underlying cause of cardiac disease is clearly distinct.

#### Fra-2

Compared to healthy controls, cardiac tissue from human SSc patients has increased levels of Fra-2, a constituent of the transcription factor AP-1 that controls a variety of stress responses such as cell proliferation, apoptosis, inflammation and wound healing (Venalis et al., 2015). In *Fra-2* transgenic mice, the *Fra-2* gene is ectopically expressed throughout the body. *Fra-2* mice develop microangiopathy (Box 1) and generalised fibrosis predominantly in the skin and lungs. This is associated with vascular remodelling, including intimal thickening, and obliteration of the pulmonary arteries (Eferl et al., 2008). Unlike in human SSc, these mice do not develop antibodies against endothelial cells or Topo I. Moreover, fibrosis still arises if B and T cells are ablated, which strongly argues against an autoimmune aetiology (Maurer et al., 2013).

*Fra-2* mice have a decreased capillary density in the myocardium and an increase in the number of apoptotic endothelial cells. Perivascular inflammation, comprising mainly T cells, and increased myocardial fibrosis are also present, accompanied by an increase in myofibroblasts (Venalis et al., 2015).

Although the aetiology of disease in *Fra-2* mice is most likely not autoimmune, this model may be useful to study the implications of increased *Fra-2* expression in SSc patient hearts.

#### Urokinase-type plasminogen activator receptor (*uPAR*)^−/−^

uPAR is widely expressed in haematopoietic and endothelial cells, fibroblasts, and myofibroblasts. It has anti-apoptotic effects and is involved in ECM remodelling and degradation, as well as cell differentiation, proliferation, adhesion and migration (Ragno, 2006). Loss of function of the uPA/uPAR system in endothelial cells is involved in SSc-related microvascular abnormalities and impaired angiogenesis (D'Alessio et al., 2004). Cleavage of uPAR is also a crucial step in the differentiation of fibroblasts into myofibroblasts (Bernstein et al., 2007), and *uPAR* gene deficiency is involved in the pathogenesis and progression of fibrosis in the skin and kidneys of mice (Zhang et al., 2002; Kanno et al., 2008). *uPAR*^−/−^ mice display the main histopathological features of human SSc, including dermal and pulmonary fibrosis, peripheral microvasculopathy and endothelial cell apoptosis. In addition, they have increased dermal thickness, collagen content, myofibroblast numbers and expression of profibrotic factors such as TGF-β (Manetti et al., 2014).

*uPAR*^−/−^ mice also exhibit the typical histopathological features of SSc cardiomyopathy, including interstitial and perivascular fibrosis (Box 1) in the myocardium, foci of cardiomyocyte damage with contraction band necrosis, endothelial cell apoptosis, reduced capillary density, profibrotic myofibroblast differentiation and patchy collagen accumulation in interstitial and perivascular areas of the myocardium. However, it has been suggested that fibrosis in these mice primarily arises as a consequence of fibroblast reprogramming, which is supported by the lack of inflammation in the myocardium (Wang et al., 2012). Despite a well-characterised cardiac phenotype, the *uPAR*^−/−^ model is therefore unlikely to be suitable for immunological questions.

## Non-rodent models of systemic autoimmunity

Rodents represent the overwhelming majority of animals used for immunological research. However, a few non-rodent models of systemic autoimmunity do exist. Recently, the susceptibility to heterogeneous collagen II for arthritis induction was confirmed in minipigs, providing the first large-animal model for RA, which might prove very valuable for future studies on cardiac pathology (Lee et al., 2016).

Interestingly, for SSc, the University of California at Davis line 200 (UCD-200) chicken is the only animal model displaying all the hallmarks of human SSc, i.e. vascular occlusion, severe perivascular lymphocytic infiltration of skin and viscera, fibrosis of skin and internal organs, ANAs, and distal polyarthritis (Wick et al., 2006). These chickens spontaneously develop an inherited scleroderma-like disease with an initial inflammatory stage, progressing to fibrosis with collagen accumulation in the affected tissues. Alterations start in the skin within the first week after hatching and then extend to internal organs including the heart. A total of 90% of the birds are afflicted at the age of 5 weeks (Sgonc and Wick, 2009).

Animal welfare and husbandry considerations, and unique opportunities for genetic manipulation and imaging techniques, have also led to a surge of immunological studies in zebrafish (Renshaw and Trede, 2012). Although the zebrafish innate immune system is now well-characterised (Novoa and Figueras, 2012), information on adaptive immunity is still scarce and models for systemic autoimmunity are not yet established.

## Summary and conclusions

Despite their significant clinical relevance, cardiac manifestations have so far rarely been the focus of experimental studies into autoimmune disease. The available literature is sparse and often limited to the first description and basic phenotypic characterisation of the respective mouse model. This means that, for most models, despite being well-established for immunological research, the mechanisms underlying cardiac involvement are not yet understood. More basic research is needed to allow direct comparison between cardiac disease mechanisms in human patients and the respective model. However, due to significant progress in our understanding of the immuno-cardio crosstalk, access to this information is becoming important to allow researchers to choose suitable models. Besides the phenotypic information provided above, we recommend that researchers also consider the following general challenges that are involved in attempting to model human disease using mice.

### 

#### Complex pathophysiology of systemic autoimmune disease

Faithful modelling of diseases with aetiologies and phenotypic manifestations as diverse as those observed in systemic autoimmunity is immensely challenging. Pathological pathways underlying organ damage in systemic autoimmunity are many-fold and interconnected, and identification of a single triggering event or molecular culprit has not been possible so far. SLE has in fact been suggested to encompass several different disease groups (Toro-Domínguez et al., 2018). This opens the possibility for patient stratification and targeted investigation of aetiology and mechanisms in specific patient groups. In the meantime, researchers commonly resort to models that phenocopy selected manifestations of disease, and the use of several different models in parallel is highly recommended.

#### Environmental factors

A common concern with mouse models is that laboratory mice are born and maintained in controlled conditions with little correspondence to the human environment (Sundberg and Schofield, 2018). This is particularly relevant for immunological studies, which are known to show strong sensitivity to changes in diet, the microbiota, or the absence or presence of environmental microbes (Mu et al., 2017; Johnson et al., 2015). Careful experimental design and confirmation experiments in separate animal facilities, which differ in standard diet and repertoire of environmental microbes, may help to avoid spurious results and limit this concern.

#### Genetic diversity

Current studies in mouse models rely heavily on inbred mouse strains; however, these cannot mimic the genetically diverse human population. Yet, variable susceptibility based on genetic variation has emerged as a critical factor determining the risk of developing autoimmunity as well as determining the main target organ in systemic autoimmunity (Almlöf et al., 2017; Lanata et al., 2018). Importantly, mouse genetic diversity panels have been generated to overcome this limitation. For example, the Collaborative Cross mouse diversity panel has been obtained from systematically crossing eight inbred founder strains (A/J, C57BL/6J, 129S1/SvImJ, NOD/ShiLtJ, NZO/H1LtJ, CAST/EiJ, PWK/PhJ and WSB/EiJ) (Iraqi et al., 2012). They provide the opportunity to identify genetic influences on manifestations of autoimmunity.

#### Inter-species differences in basic physiology

While cardiovascular system proteins, including myosin and troponin, are highly conserved, the immune system is under high evolutionary pressure from pathogens (Fumagalli et al., 2011). Therefore, mouse and human immunological factors may differ, which may affect direct translatability from model to patients. However, while individual molecules may have changed throughout evolution, the overall function of cells and networks often remains the same (Monaco et al., 2015). Thus, a specific target identified in mouse models needs to be carefully validated in humans, but it is likely that the functional pathways involved are similar.
